# Supplementary material for: ZRT1 Harbors an Excess of Nonsynonymous Polymorphism and Shows Evidence of Balancing Selection in Saccharomyces cerevisiae
Source: G3 (Bethesda). 2013 Apr 1;3(4):665–73. doi: 10.1534/g3.112.005082 (PMC3618353; doi:10.1534/g3.112.005082)
Supplement: Supporting Information [file supp_3_4_665__index.html]

ZRT1 Harbors an Excess of Nonsynonymous Polymorphism and Shows Evidence of Balancing Selection in Saccharomyces cerevisiae — Supporting Information 

# *ZRT1* Harbors an Excess of Nonsynonymous Polymorphism and Shows Evidence of Balancing Selection in *Saccharomyces cerevisiae*

## Supporting Information for Engle and Fay, 2013

**Files in this Data Supplement:**

- Supporting Information - Figures S1 and S2, File S1, and Tables S1-S3 (PDF, 151 KB)
- Figure S1 - Neighbor-joining tree of 21 concatenated control genes (PDF, 83 KB)
- Figure S2 - Neighbor-joining tree of *ZRT1* and adjacent genes *ADH4* and *FZF1* (PDF, 100 KB)
- File S1 - Final Dataset (.zip, 128 KB)
- Table S1 - Genome sequences used in this study (.xlsx, 10 KB)
- Table S2 - Polymorphism and divergence data (.xlsx, 14 KB)
- Table S3 - Maximum likelihood HKA test results (.xlsx, 9 KB)
