# Supplementary material for: ZRT1 Harbors an Excess of Nonsynonymous Polymorphism and Shows Evidence of Balancing Selection in Saccharomyces cerevisiae
Source: G3 (Bethesda). 2013 Apr 1;3(4):665–73. doi: 10.1534/g3.112.005082 (PMC3618353; doi:10.1534/g3.112.005082)
Supplement: Supporting Information [file supp_g3.112.005082_005082SI.pdf]

G3 · Genes | Genomes | Genetics

ZRT1 harbors an excess of nonsynonymous polymorphism and shows evidence of balancing selection in *Saccharomyces cerevisiae*

Elizabeth K. Engle[*] and Justin C. Fay[§]

[*]Molecular Genetics and Genomics Program, Washington University, St. Louis, MO, 63108
[§]Department of Genetics and Center for Genome Sciences and Systems Biology, Washington University, St. Louis, MO, 63108

DOI: 10.1534/g3.112.005082

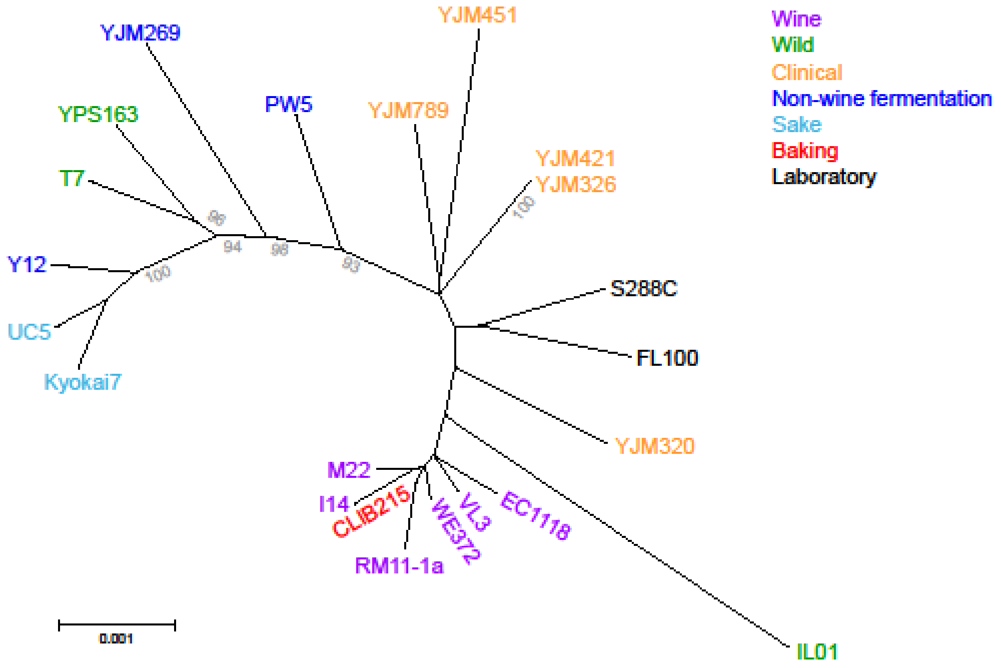

Figure S1 Neighbor-joining tree of 21 concatenated control genes. An unrooted neighbor-joining tree of the concatenated 21 control genes along with bootstrap values greater than 90% (in gray). Only strains used in the *ZRT1* tree are shown. *S. cerevisiae* strains are color coded by strain class (see legend).

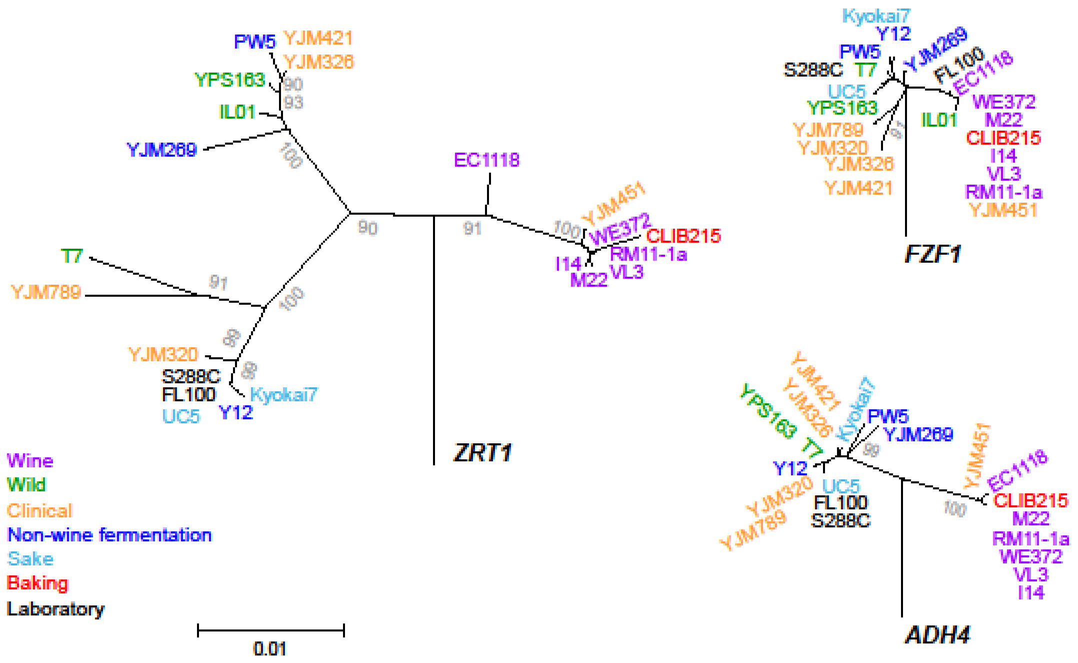

Figure S2 Neighbor-joining tree of *ZRT1* and adjacent genes *ADH4* and *FZF1*. Neighbor-joining trees of *ZRT1* and the two adjacent genes *ADH4* and *FZF1* with bootstrap values greater than 90% (in gray) and rooted to *S. paradoxus*. *S. cerevisiae* strains are color coded by class (see legend). The strains represented in each tree are identical except strain IL01 is missing for the gene *ADH4*.

File S1

Final Dataset

File S1 is available for download at http://www.g3journal.org/lookup/suppl/doi:10.1534/g3.112.005082/-/DC1.

Tables S1-S3 are available for download at http://www.g3journal.org/lookup/suppl/doi:10.1534/g3.112.005082/-/DC1.

Table S1 Genome sequences used in this study

Table S2 Polymorphism and divergence data

Table S3 Maximum likelihood HKA test results
