# Supplementary material for: ZRT1 Harbors an Excess of Nonsynonymous Polymorphism and Shows Evidence of Balancing Selection in Saccharomyces cerevisiae
Source: G3 (Bethesda). 2013 Apr 1;3(4):665–73. doi: 10.1534/g3.112.005082 (PMC3618353; doi:10.1534/g3.112.005082)
Supplement: Supporting Information [file supp_g3.112.005082_FigureS1.pdf]

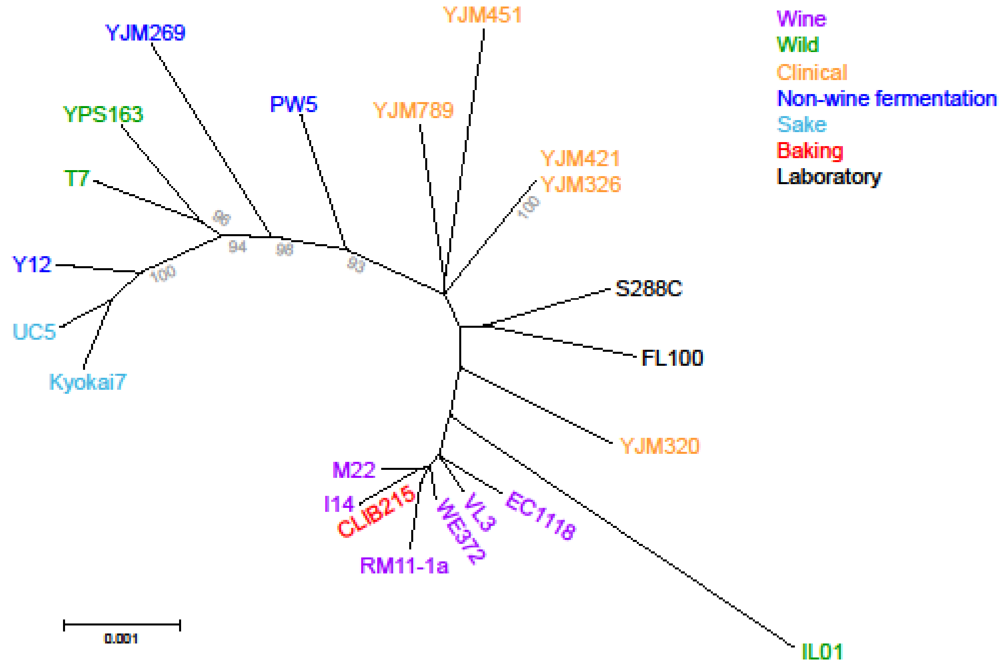

Figure S1 Neighbor-joining tree of 21 concatenated control genes. An unrooted neighbor-joining tree of the concatenated 21 control genes along with bootstrap values greater than 90% (in gray). Only strains used in the *ZRT1* tree are shown. *S. cerevisiae* strains are color coded by strain class (see legend).
