# Supplementary material for: ZRT1 Harbors an Excess of Nonsynonymous Polymorphism and Shows Evidence of Balancing Selection in Saccharomyces cerevisiae
Source: G3 (Bethesda). 2013 Apr 1;3(4):665–73. doi: 10.1534/g3.112.005082 (PMC3618353; doi:10.1534/g3.112.005082)
Supplement: Supporting Information [file supp_g3.112.005082_FigureS2.pdf]

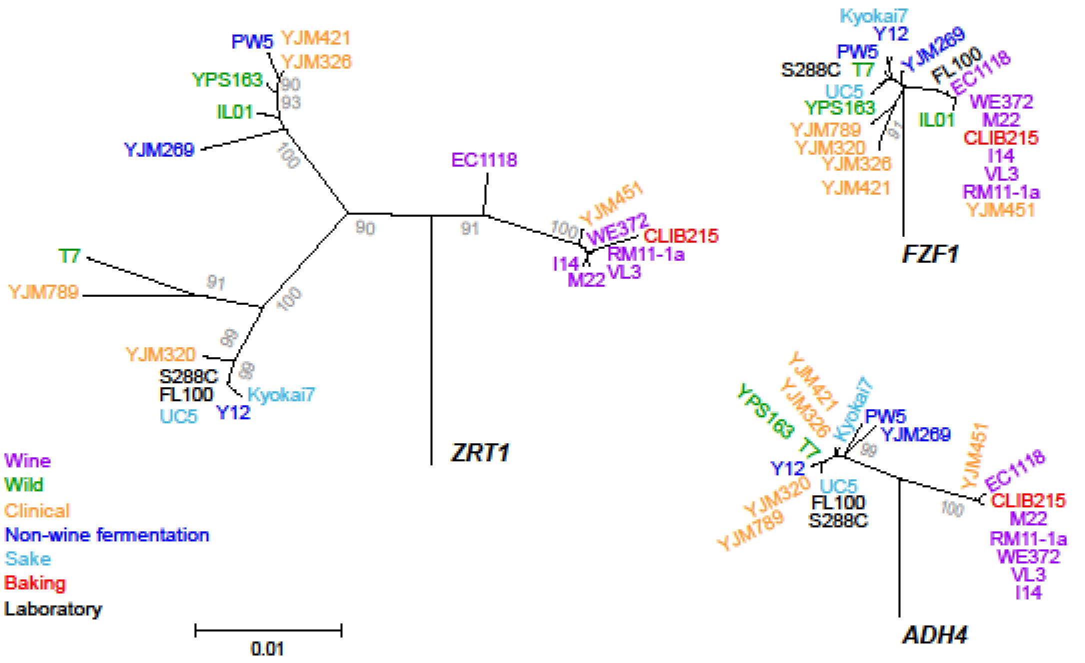

Figure S2 Neighbor-joining tree of *ZRT1* and adjacent genes *ADH4* and *FZF1*. Neighbor-joining trees of *ZRT1* and the two adjacent genes *ADH4* and *FZF1* with bootstrap values greater than 90% (in gray) and rooted to *S. paradoxus*. *S. cerevisiae* strains are color coded by class (see legend). The strains represented in each tree are identical except strain IL01 is missing for the gene *ADH4*.
